# An open label phase II study of safety and clinical activity of naltrexone for treatment of hormone refractory metastatic breast cancer

**DOI:** 10.1007/s10637-022-01317-4

**Published:** 2022-11-28

**Authors:** Jayanthi Vijayakumar, Tufia Haddad, Kalpna Gupta, Janet Sauers, Douglas Yee

**Affiliations:** 1 Division of Hematology Oncology and Transplantation Department of Medicine, University of Minnesota, MN Minneapolis, USA; 2 Mayo Clinic Cancer Center, MN Rochester, USA; 3 Division of Hematology/Oncology, Department of Medicine, University of California, CA Irvine, USA; 4 Masonic Cancer Center, University of Minnesota, Minneapolis, MN USA; 5 420 Delaware Street SE, Minneapolis, MN 55455 USA

**Keywords:** Breast cancer, Estrogen receptor, Mu-opioid receptor, Naltrexone, Positron emission tomography

## Abstract

The opioid receptor (OR) antagonist naltrexone inhibits estrogen receptor-α (ER) function in model systems. The goal of this study was to determine the clinical activity of naltrexone in patients with ER-positive metastatic breast cancer. Patients with hormone receptor positive metastatic breast cancer were enrolled on a phase II study of naltrexone. An escalating dose scheme was used to reach the planned dose of 50 mg daily. The primary objective of the study was to evaluate response to therapy as measured by stabilization or reduction of the tumor Maximum Standardized Uptake Value (SUVmax) at 4 weeks by PET-CT scan. The secondary objectives included safety assessment and tumor SUVmax at 8 weeks. Out of 13 patients we enrolled, 8 patients had serial PET-CT scans that were evaluable for response. Of these 8 patients, 5 had stable or decreased SUVmax values at 4 weeks and 3 had clinical or imaging progression. Median time to progression was short at 7 weeks. Naltrexone was well tolerated. There were no discontinuations due to toxicity and no grade 3 or 4 toxicities were noted. Naltrexone showed modest activity in this short study suggesting the contribution of opioid receptors in ER-positive breast cancer. Our data do not support further development of naltrexone in hormone refractory breast cancer. It is possible that more potent peripherally acting OR antagonists may have a greater effect. (*ClinicalTrials.gov Identifier: NCT00379197 September 21, 2006*).

## Introduction

Approximately 72% of breast cancers diagnosed are hormone receptor positive and HER-2 neu negative [1]. While targeting of estrogen receptor-α (ER) remains the mainstay of treatment for advanced hormone receptor positive breast cancers, tumor resistance to this strategy remains a challenge. Combination of endocrine therapy with mTOR inhibitors and CDK4/6 inhibitors has been a major advancement in the setting of endocrine resistance [2, 3]. The success of these strategies shows how improving estrogen receptor targeting improves outcomes for women with advanced breast cancer. Further, interest in developing new oral agents to disrupt ER function are ongoing [4]. Additional strategies to disrupt this signaling pathway could improve patient outcomes.

Morphine, which is a µ-opioid receptor (MOR) agonist, promotes angiogenesis-dependent growth in estrogen-dependent human MCF-7 cell tumor xenografts in mice at physiologically relevant conditions [5]. The MOR antagonist, naloxone, inhibited growth of these cells [6] by acting as a selective estrogen receptor modulator. Thus, MOR antagonists may inhibit hormone receptor positive tumor growth by inhibiting angiogenesis and targeting the ER signaling pathway.

Naltrexone is an opioid antagonist that is eight times more active and three times longer acting as naloxone, and previously has been shown to have anti-tumor activity in neuroblastoma-inoculated mice [7]. Mammary tumors in mice (established with 7,12-dimethylbenzanthracene) had 70% response rate and 23% reduction in tumor size with oral administration of naltrexone. There was correlation between tumor regression and presence of ER and progesterone receptor (PR) in the tumor tissue specimens [8].

Naltrexone has also been studied in other tumor types in humans. Malignant astrocytoma patients treated with radiotherapy and oral naltrexone survived longer than patients treated with radiotherapy alone [9]. In patients with metastatic renal cell carcinoma refractory to IL-2 monotherapy, the addition of naltrexone at 100 mg every other day demonstrated 60% response rate as defined by non-progressive disease [10]. Preclinical studies in ovarian and colon cancer studied naltrexone and methylnatrexone respectively along with standard chemotherapeutic agents (platinum and 5-fluorouracil) [11, 12] with promising results including suppression of cellular proliferation and in vivo tumor progression. In patients with advanced cancer and opioid-induced constipation randomized to methylnaltrexone and placebo, an unplanned post hoc analysis revealed that treatment with methylnaltrexone was associated with improved overall survival [13].

The effects of naltrexone in breast cancer physiology are unknown. The purpose of this study was to determine the safety and efficacy of naltrexone for the treatment of metastatic breast cancer that is refractory to hormonal therapy.

## Methods

This single center open label phase II study was conducted at the M Health Fairview Masonic Cancer Clinic. Informed consent was obtained prior to performance of any study related procedures or assessments. The study was reviewed and protocol approved by institutional review board. The study was funded by a supplement to the Masonic Cancer Center’s Cancer Center Support Grant (P30 CA 077598) and registered as clinical trials number NCT00379197. Naltrexone was purchased for use in this trial.

### Objectives

Previous work showed that PET-CT can predict early response to endocrine therapy [14, 15]. To rapidly determine the activity of naltrexone in patients with advanced breast cancer, PET-CT SUVmax was utilized as a surrogate marker of response at 4 and 8 weeks.

The primary objective of the study was to evaluate the efficacy of naltrexone for the treatment of hormone-refractory, metastatic breast cancer as measured by [18 F] fluorodeoxyglucose (FDG) PET-CT SUVmax after 4 weeks. The secondary objectives were to evaluate the safety of naltrexone in the treatment of hormone-refractory, metastatic breast cancer (as assessed by CTCAE version 3.0), tumor response by SUVmax at 8 weeks, and to determine the median time to event (first time when SUVmax is higher than that at baseline) within one year from enrollment.

### Patient selection

The following subjects were eligible for this study: diagnosis of metastatic hormone receptor positive (ER and/or PR positive) breast cancer, 18 years of age or greater, disease progression after prior endocrine therapy, and Karnofsky performance status > 70% (ECOG 0–1). All subjects were required to have adequate bone marrow reserve (absolute neutrophil count ≥ 1.5 × 10^9^/L, platelets > 100 × 10^9^/L, hemoglobin > 9 g/L), adequate hepatic function (bilirubin < 2x upper limit of normal [ULN], aspartate transaminase < 2x ULN) and renal function (creatinine < 2x ULN). Women of child-bearing potential were required to use an effective method of contraception (i.e., a hormonal contraceptive, intra-uterine device, diaphragm with spermicide, condom with spermicide or abstinence) during the study and for 3 months after the last dose of study drug. Subjects were required to have terminated prior endocrine treatment at least 2 weeks prior to study enrollment. Prior chemotherapy, immunotherapy or biological therapy was allowed provided there was at least three weeks since last treatment and patient had recovered from effects of the treatment. Subjects were required to have measurable disease defined by RECIST 1.0 criteria. All subjects provided voluntary written informed consent.

Patients were excluded from participation in the study if they were pregnant or lactating, had brain metastasis (unless stable for 1 month or more following radiation therapy), were on short-acting or long-acting opioid medication (including morphine, hydromorphone, hydrocodone, fentanyl, tramadol) or had uncontrollable pain with the use of non-narcotic drugs (acetaminophen or non-steroidal medications). Patients on immunosuppressive therapy for autoimmune diseases, organ transplant or indications were also excluded from the study.

### Treatment intervention

Naltrexone was administered to patients daily in escalating doses. Study subjects were given 10 mg of naltrexone orally once daily for one week, then 25 mg daily for one week, and then further increased to a maximum of 50 mg daily as tolerated. Naltrexone was held for any grade 3 or 4 toxicities. Once the toxicity had resolved, the therapy was resumed at the next lower dose, with dose reductions as follows: 50 to 25 mg daily, 25 to 10 mg daily, 10 mg to 5 mg daily or 5 mg daily to every other day.

### Study design and statistical plan

Patients had physical exam, assessment of toxicity and performance status, as well as tumor assessment by PET-CT imaging, at the beginning of study, after 4 weeks, 8 weeks, and then every 8 weeks thereafter. Efficacy was evaluated by SUVmax of a metastatic lesion by 18 F-FDG PET-CT imaging, a surrogate marker of tumor response. An objective response was recorded if the tumor demonstrated a decrease in FDG uptake (SUVmax) from baseline by 50% or greater in at least one of the metastatic sites as measured by PET-CT imaging. Stable disease was defined as SUVmax maintained at or decreased by < 50% from the baseline level. RECIST 1.1 was used to evaluate objective responses [16].

Patients with objective response to therapy or disease stabilization by PET-CT SUVmax were allowed to continue treatment on study after the initial 4-week assessment. Treatment and study participation was discontinued if the patient developed pain that required narcotics, disease progression as noted by increasing SUVmax or presence of new metastasis, grade 3 or 4 toxicities that do not resolve within 4 weeks of holding therapy, did not tolerate dose reduction to 5 mg every other day, became pregnant or failed to use adequate birth control or if the treating physician decided to change therapy in the best interest of the patient. Patients were also followed for overall survival.

A Simon two-stage phase II design [17] was utilized to test the null hypothesis that response rates was p ≤ 0.10 versus the alternative that p ≥ 0.30. After enrolling 19 patients in the first stage, the trial would be terminated if fewer than 3 patients responded, as defined by any decline in SUVmax. If trial continued to the second stage, a total of 34 patients was planned to be enrolled. If the total number responding was less than or equal to 6, the treatment would be rejected. This design attained a power of 0.90 and has 0.04 alpha error rate. However due to slow accrual and approval of new endocrine therapies in breast cancer (everolimus, palbociclib) the study was terminated early.

## Results

A total of 13 patients were enrolled in the study between 2006 and 2013. Eight patients were evaluable for the primary endpoint having completed two consecutive PET-CT scans necessary to evaluate tumor response. Five patients were not evaluable for the following reasons: never took naltrexone, significant pain which needed opioid use after consent of study, naltrexone discontinued after the first dose due to side effect (hot flash), or never followed up after study consent.

### Patient characteristics

Of the 8 evaluable patients 50% were below 65 years of age with a range of 50–90 years. All patients had experienced disease progression on prior endocrine therapy and received a median of 1 prior chemotherapy regimen for metastatic disease. Patient characteristics are outlined in Table 1.

**Table 1:** Patient Demographics and Baseline Characteristics

	N = 8
Age: mean (range) years	68 (50–90)
Gender	
Male Female	(0/8) (8/8) 100%
Race/Ethnicity	
Non-Hispanic White African American Asian	(8/8) 100% (0/8) (0/8)
Brain metastasis	(1/8) 12.5%
Prior therapy for diagnosis of metastatic disease	
Median (range) Prior endocrine therapy regimens	2 (1–3) 1 (0–12)
Prior chemotherapy regimens	

### Tumor response by PET-CT assessment

**Fig. 1:**
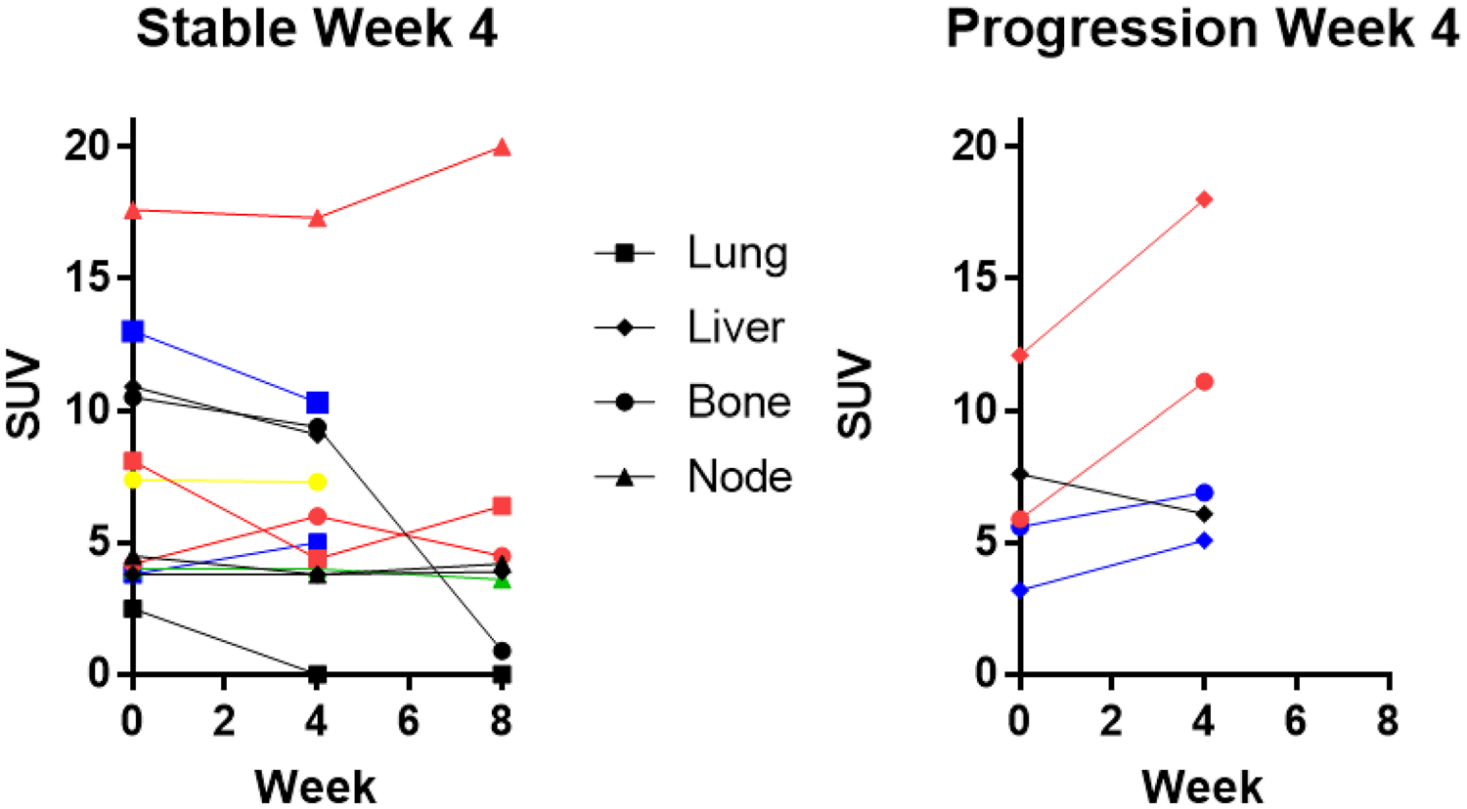
Tumor response by SUVmax after 4 and 8 weeks of therapy. Each individual patient is represented by a separate color. If one metastatic site decreased at week 4, the patient was considered to have stable disease. One patient was deemed to have progression (black) due to development of new sites at week 4

Of the 8 evaluable patients, five were determined to have stable or decreased SUVmax values after 4 weeks of naltrexone (Fig. 1). Two patients had elevated SUVmax values after 4 weeks of naltrexone indicative of progression, and one patient who progressed due to the presence of a new metastatic lesion had a decrease in SUVmax at the index site (as indicated in Fig. 1 right panel, black line).

Since patients had multiple sites of metastasis, each individual organ site was measured. The patient who had a partial response had a decrease in SUVmax of > 50% at one site of metastasis, however the rest of the metastatic sites had a decrease in SUVmax of < 50%.

Among the 8 patients, 3 patients had lung metastasis. There was an overall decrease in SUVmax in the lung metastasis compared to other sites of metastasis in the 3 patients. Mean percentage decrease in SUVmax for lung metastasis among the three patients was 56%.

### Other clinical efficacy endpoints

By RECIST criteria, one patient had a partial response in a liver lesion after 8 weeks of therapy. This was accompanied by a decrease in SUVmax below baseline. However, after 12 weeks of therapy there was evidence of disease progression, there were no patients who had clinical benefit as defined by stable disease for more than 24 weeks.

**Fig. 2:**
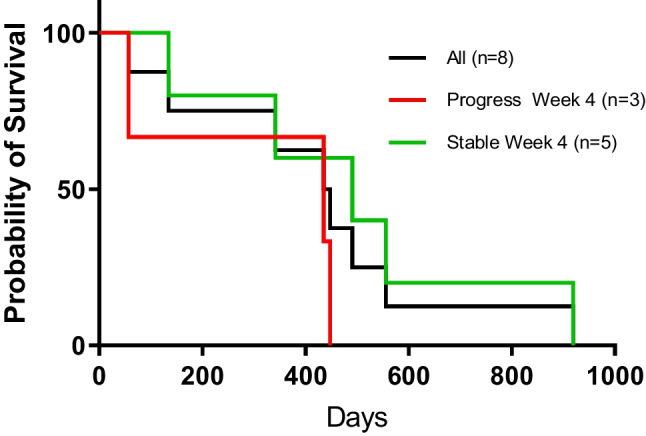
Overall Survival by Kaplan-Meier estimate (days) of study patients

The median time to tumor progression for the remaining 7 participants was 8 weeks. No patients needed to discontinue study therapy because of a requirement for opioid narcotics. Therapy was discontinued because of tumor progression in all cases. The overall survival of patients is as shown in Fig. 2.

### Safety

There were no grade 3 or 4 serious adverse events in this study. However four out of eight patients had one of the following adverse events (regardless of attribution to naltrexone), including weight gain, myalgia, insomnia, dyspnea, pruritus, palmar-planter erythrodesstheia syndrome, hot flashes, anemia, ascites, bloating, nausea, diarrhea, and elevated AST. Nausea, abdominal bloating, diarrhea, and elevated AST were at least possibly attributed to study drug. One patient discontinued therapy for grade 2 hot flashes, but there were no discontinuations of naltrexone due to grade 3 or 4 toxicities. One patient chose to discontinue therapy for grade 2 hot flashes.

## Discussion

This is the first clinical trial evaluating the efficacy of naltrexone in breast cancer. Naltrexone was well tolerated in women heavily pretreated with endocrine therapy and/or chemotherapy for hormone receptor positive advanced breast cancer. Even though there were few patients in study, there was a partial response observed in one of eight evaluable patients after 8 weeks of treatment, while stable disease was seen in an additional 4 patients. Expansion of the cohort might have provided better insight into the efficacy of the drug; however, we decided to close this study prior to meeting accrual goals given the slow recruitment. This was due to the approval of several new drugs effective for endocrine resistant breast cancer (mTOR and CDK4/6 inhibitors) emerging during the conduct of this study. It is possible that initiating naltrexone treatment earlier or in combination of the other targeted agents might provide greater benefit.

Opioid receptor antagonists have shown statistically significant single agent activity in preclinical models of breast cancer, and they have also been examined in combination with other systemic therapies [18]. Several peripherally acting mu opioid receptor agonists (PAMORAs) have been approved by the FDA for opioid-induced constipation (OIC), including methylnaltrexone (MNTX). MNTX has been shown to potentiate effects of mTOR inhibitors [19] and function synergistically. Treatment with MNTX or silencing of MOR inhibited Lewis lung carcinoma (LLC) invasion and anchorage-dependent growth as well as significantly reduced LLC growth and metastases in mice [20]. Notably, in advanced malignancies MNTX used for OIC showed improved survival upon post-hoc analysis [13]. In human gastric cancer cell xenografted mice treated with docetaxel, co-treatment with MNTX led to prolonged survival and significantly reduced metastasis compared to docetaxel alone [21]. It is likely that PAMORAs have a greater effect than naltrexone and may not antagonize analgesia because of their inability to cross the blood brain barrier. In the BOLERO-2 study, the mTOR inhibitor everolimus in combination with exemestane showed significant improvement in progression-free survival compared to exemestane alone [22]; however, everolimus was associated with significant toxicity [23]. If drugs like MNTX work in a similar fashion to inhibit mTOR, it would be attractive to develop strategies where enhanced benefit with reduced toxicity could be employed.

Additional oral targeted therapies have been approved for treatment of endocrine resistant metastatic breast cancer that are highly effective [24], and in the case of alpelisib, are linked to specific mutations in the PIK3CA gene [25]. While naltrexone was effective in stabilizing the tumor SUVmax in most patients over a short interval, the median time to progression and overall survival in this study cohort was short. A related strategy using the MOR antagonist naloxegol is being tested in lung cancer (NCT03087708). Our study provides a proof of principle that opioid receptor antagonists have the potential to improve treatment outcomes in breast cancer. It is likely that using more potent OR antagonists and starting earlier during disease progression may have improve outcomes of standard therapies.

## Data Availability

Anonymized data will be made available.
